# Health-Related Quality of Life, Anxiety, and Stress in Women with Uterine Fibroids: A Cross-Sectional Analysis

**DOI:** 10.3390/jcm15124777

**Published:** 2026-06-19

**Authors:** Agnieszka Lach, Wiktoria Jędrzejak, Patrycja Loba, Maria Depczyńska, Zuzanna Radziszewska, Dobrochna Stachecka, Maciej Wilczak, Karolina Chmaj-Wierzchowska

**Affiliations:** 1 Department of Maternal and Child Health and Minimally Invasive Gynecologic Surgery, Poznan University of Medical Sciences, 60-701 Poznan, Poland; 2 Faculty of Medicine, Poznan University of Medical Sciences, 60-701 Poznan, Polandpatrycja.loba@outlook.com (P.L.);; 3 Laboratory of Advanced Interventional Therapies in Gynecology and Urogynecology, Poznan University of Medical Sciences, 60-701 Poznan, Poland

**Keywords:** uterine fibroids, leiomyoma, health-related quality of life, HRQL, anxiety, stress, UFS-QoL, APAIS, women’s health, gynecology

## Abstract

**Background**: Uterine fibroids are among the most common benign tumors affecting women of reproductive age and may substantially impair health-related quality of life (HRQL). Although anxiety and stress are frequently reported by affected women, their contribution to HRQL remains unclear. This study aimed to evaluate the relationships between symptom severity, anxiety, stress, and HRQL in women with uterine fibroids. **Methods**: A cross-sectional study was conducted among 107 women hospitalized for uterine fibroid treatment. Symptom severity and HRQL were assessed using the Uterine Fibroid Symptom and Quality of Life (UFS-QoL) questionnaire. Anxiety and information needs were evaluated using the Amsterdam Preoperative Anxiety and Information Scale (APAIS), while subjective anxiety and stress levels were measured with the Visual Analog Scale (VAS). Associations between variables were analyzed using non-parametric tests, Spearman’s correlations, and multiple regression analysis. **Results**: Clinically significant anxiety was observed in 41.1% of participants. The mean HRQL score was 57.4 ± 22.3 points. In multivariate analysis, symptom severity was the only independent predictor of HRQL (β = −0.67, *p* < 0.001), explaining approximately 45% of its variance. Anxiety, stress, and sociodemographic factors were not independently associated with overall HRQL. However, higher levels of anxiety and stress were significantly associated with poorer sexual functioning. Women living in rural areas and those with higher body weight reported poorer outcomes in selected quality-of-life domains. **Conclusions**: Symptom severity is the primary determinant of HRQL in women with uterine fibroids. Although anxiety and stress do not independently predict overall quality of life, they may adversely affect sexual functioning. These findings support a comprehensive management approach that combines symptom-oriented treatment with psychological and educational support.

## 1. Introduction

Uterine fibroids (leiomyomas) are among the most common benign tumors affecting women of reproductive age and constitute a major public health concern [1,2]. In addition to causing physical symptoms, uterine fibroids substantially impair health-related quality of life (HRQL) and daily functioning [1,3]. Their clinical presentation is heterogeneous and most commonly includes heavy menstrual bleeding, chronic pelvic pain, and pressure-related symptoms [4,5]. These manifestations may reduce physical activity, decrease work productivity, and negatively affect social and sexual functioning. As a result, uterine fibroids may significantly impair emotional well-being, self-esteem, and interpersonal relationships, making HRQL an important outcome measure in gynecological care [1,3]. Beyond the somatic burden of the disease, increasing attention has been directed toward its psychological consequences. Women with uterine fibroids frequently report elevated levels of anxiety and stress, particularly in the preoperative period [6]. Anxiety associated with planned surgical treatment may arise from concerns regarding anesthesia, the procedure itself, potential complications, and the impact of treatment on bodily integrity and reproductive health [6,7]. Importantly, previous studies suggest that preoperative anxiety does not necessarily correspond to the clinical severity of symptoms [7]. Psychological distress may also be intensified by uncertainty and insufficient medical information.

Although patient-reported outcomes are increasingly recognized as important clinical indicators, the relationships between symptom severity, preoperative anxiety, stress, and health-related quality of life (HRQL) in women with uterine fibroids remain insufficiently understood. In particular, it is unclear whether impaired quality of life is primarily driven by the severity of fibroid-related symptoms or by the psychological burden associated with awaiting surgical treatment. Therefore, the present study aimed to evaluate symptom severity, HRQL, preoperative anxiety, stress, and information needs among women hospitalized for planned surgical treatment of uterine fibroids. Using standardized assessment tools, including the UFS-QoL questionnaire, the Amsterdam Preoperative Anxiety and Information Scale (APAIS), and the Visual Analog Scale (VAS), we investigated the relationships between clinical symptoms, psychological factors, and quality of life. We also examined whether psychological factors were associated with reduced quality of life in specific functional domains and whether symptom severity predicted anxiety levels. We hypothesized that both symptom severity and psychological burden would contribute to reduced HRQL in women with uterine fibroids.

## 2. Materials and Methods

This cross-sectional survey study included 107 women diagnosed with uterine fibroids who were hospitalized for planned surgical treatment at the Department of Maternal and Child Health and Minimally Invasive Gynecologic Surgery, Heliodor Święcicki Clinical Hospital of the Poznan University of Medical Sciences, between October 2025 and March 2026. Eligibility criteria included age ≥ 18 years, a confirmed diagnosis of uterine fibroids based on clinical and/or imaging findings, qualification for surgical treatment, and the provision of written informed consent. Women with incomplete questionnaire data or those who declined participation were excluded from the study. Because all participants were awaiting planned surgical treatment, preoperative anxiety and information needs were assessed using the Amsterdam Preoperative Anxiety and Information Scale (APAIS) [6]. Patients’ perceived psychological burden. The study questionnaire consists of six items rated on a 5-point Likert scale (1 = not at all to 5 = extremely). Four items assess anxiety related to anesthesia and the planned surgical procedure, whereas two items evaluate the patient’s need for medical information. The total anxiety score ranges from 4 to 20 points, with scores ≥11 indicating clinically significant preoperative anxiety. Higher scores reflect greater levels of anxiety and informational needs. The APAIS is widely used for the assessment of preoperative psychological status and has demonstrated good validity and reliability in surgical populations. In addition, the Visual Analog Scale (VAS) enables the assessment of subjective anxiety and stress levels, providing complementary information regarding patients’ emotional status [6,7]. Participants were asked to rate their current level of anxiety and stress on separate 10-point scales, where 0 indicated no anxiety/stress, and 10 indicated the highest imaginable level of anxiety/stress. The VAS is a simple and widely used instrument for the assessment of subjective emotional states and provides complementary information regarding patients’ perceived psychological burden. The study questionnaire consisted of sociodemographic and clinical sections as well as standardized assessment instruments. Symptom severity and health-related quality of life (HRQL) were assessed using the Uterine Fibroid Symptom and Quality of Life (UFS-QoL) questionnaire, which evaluates six domains of quality of life: concern, activities, mood and energy, control, self-consciousness, and sexual functioning [8,9,10].

According to Polish regulations, this questionnaire-based observational study did not constitute a medical experiment and therefore did not require formal ethical approval. The study protocol was reviewed by the Bioethics Committee of Poznan University of Medical Sciences, which confirmed that the project was not a medical experiment and issued a positive opinion (No. 278/25, 23 April 2025). The study was conducted in accordance with the principles of the Declaration of Helsinki and Good Clinical Practice (GCP). All participants provided informed consent prior to participation.

### Data Analysis

For comparative analyses, selected categorical variables were recategorized into broader groups to ensure adequate subgroup sizes and improve statistical interpretability.

Statistical analyses were performed using Statistica version 14 (Cloud Software Group, Inc., Palo Alto, CA, USA), Jamovi version 2.3, and Microsoft Excel (Microsoft Office 2019, version 2205).

The distribution of variables was assessed using the Shapiro–Wilk test. Comparative analyses were performed using the Mann–Whitney U test, with effect sizes estimated using the rank-biserial correlation coefficient. Associations between categorical variables were analyzed using Pearson’s chi-square test or Fisher’s exact test, as appropriate.

For bivariate analyses, odds ratios (ORs) with 95% confidence intervals (CIs) were calculated, and the strength of associations was evaluated using the φ coefficient and Cramér’s V. Correlations between anxiety, stress, symptom severity, and quality of life were assessed using Spearman’s rank correlation coefficient (ρ).

Multivariate analysis was performed using a general linear model (GLM). Multicollinearity was evaluated using variance inflation factors (VIFs), and residual distributions were examined to verify model assumptions. Effect sizes were expressed using partial eta-squared (η^2^). A *p*-value < 0.05 was considered statistically significant.

## 3. Results

### 3.1. Characteristics of the Study Group

The mean age of the respondents was 44.88 ± 5.98 years (range: 29–61 years), with a median age of 45 years. The mean body weight was 68.59 ± 10.94 kg, and the mean body mass index (BMI) was 24 ± 3.55, indicating that most participants had a normal body weight. Table 1 shows the anthropometric measurements of the respondents.

The study group predominantly included married women (66.36%) and those with higher education (60.75%). Most participants lived in urban areas, and the most frequently reported type of occupation was non-manual work (73.83%). Regarding nutritional status, 66.36% of women reported normal body weight, 27.10% were overweight, 5.61% were obese, and 0.93% were underweight. Table 2 shows the characteristics of the study group.

Nearly half of the women in the study had not given birth (47.66%), and most of them had not experienced miscarriages (86.92%) or had undergone gynecological surgery (76.70%). Table 3 shows the characteristics of the study group in terms of obstetric history.

The mean duration of menstruation was 5.65 ± 1.42 days (range: 4–14 days). Menstrual blood loss was rated at a mean of 4.93 ± 2.2 points on a 0–10 scale. The most frequently reported clinical symptoms were dysmenorrhea (30.84%), dyspareunia (17.76%), and pain during defecation (18.69%). Daily pain complaints were reported by 14.02% of the respondents, while pain during urination occurred sporadically (0.93%). Table 4 shows the additional clinical symptoms of the study participants.

### 3.2. Anxiety, Stress, and Need for Information–APAIS

Based on the APAIS questionnaire, the total anxiety score ranged from 5 to 20 points, with a mean value of 9.67 ± 2.98 (95% CI: 9.10–10.24). Clinically significant anxiety was identified in 44 participants (41.12%), whereas 63 women (58.88%) had anxiety levels within the normal range.

The mean anxiety score related to anesthesia was 4.57 ± 1.64, while anxiety related to the planned surgical procedure reached 5.10 ± 1.62. The mean information-need score was 6.01 ± 1.96 (95% CI: 5.63–6.39). Detailed APAIS results are presented in Table 5 and Table 6.

### 3.3. Anxiety and Stress—VAS Self-Assessment

Self-assessed anxiety measured using the VAS ranged from 0 to 10 points, with a mean score of 4.66 ± 2.22 (95% CI: 4.24–5.09). Self-assessed stress also ranged from 0 to 10 points, with a mean value of 4.82 ± 2.19 (95% CI: 4.40–5.24). Overall, participants reported moderate levels of anxiety and stress.

The study did not reveal significant associations between place of residence and levels of anxiety, stress, or information needs assessed using VAS (Table 7).

Urban residents tended to report higher anxiety levels, particularly anxiety related to anesthesia; however, these differences did not reach statistical significance (Table 8).

No significant associations were observed between patients’ age, education, marital status, body weight, or history of childbirth and levels of anxiety, stress, and need for information (APAIS I–IV) as well as self-assessed anxiety and stress (VAS V–VI). Furthermore, no associations were found between anxiety assessment (APAIS I–III) and age, education, place of residence, marital status, body weight, or history of childbirth among the participants (Table 9).

No significant associations were observed between the level of information need assessed (Table 10) using the APAIS scale and the analyzed sociodemographic factors, including age, education, place of residence, marital status, body weight status, and history of childbirth (*p* > 0.05).

### 3.4. Comparative Analysis

Symptom severity ranged from 0% to 84.38% of the total score, with a mean value of 48.60 ± 20.97 points (95% CI: 44.58–52.62). Descriptive statistics are presented in Table 11.

Quality-of-life scores across individual UFS-QoL domains are summarized in Table 12. The highest mean scores were observed for mood/energy (60.35 ± 22.44) and sense of control (59.95 ± 23.25), whereas the lowest scores were reported for concerns (51.92 ± 29.67) and sexual functioning (51.52 ± 28.30).

Overall HRQL scores ranged from 6.03% to 100%, with a mean value of 57.41 ± 22.30 (95% CI: 53.14–61.69). Descriptive statistics for HRQL are presented in Table 13.

Correlation analysis revealed no significant associations between anxiety, stress, information needs, symptom severity, and overall HRQL (Table 14). The only significant findings concerned the sexual functioning domain, which was negatively correlated with self-reported anxiety and stress.

No significant associations were observed between age and symptom severity or quality of life (Table 15).

Women with higher education demonstrated significantly better quality of life in the sexual functioning domain than women with lower educational attainment (Table 16). A similar trend was observed in the activities domain, although the difference did not reach statistical significance.

Place of residence was significantly associated with symptom severity and several HRQL domains (Table 17). Women living in rural areas reported greater symptom severity and lower quality of life compared with urban residents. The only exception was the self-awareness domain, which showed a non-significant trend toward poorer outcomes among rural residents.

No significant associations were found between marital status and symptom severity or quality of life (Table 18).

Participants with normal body weight demonstrated significantly better outcomes in the domains of concerns and sexual functioning than overweight participants (Table 19). Similar trends were observed for activities and self-awareness, although these differences did not reach statistical significance.

Likewise, childbirth history was not associated with symptom severity, HRQL, or any of the analyzed quality-of-life domains (Table 20).

### 3.5. Regression Model

To identify the independent determinants of HRQL, we conducted a multiple regression analysis using a GLM. The model included clinical variables (symptom severity, anxiety level, self-assessed stress, and need for information) and sociodemographic variables (age, education, place of residence, marital status, history of childbirth, and BMI). While a comprehensive range of factors was examined from a substantive perspective, their number was intentionally limited to prevent the artificial inflation of standard errors, thereby preserving the statistical power of the tests. The final model structure, comprising 10 predictors with a sample size of 107 participants, was selected to maintain statistical rigor while adequately addressing the key theoretical determinants of the studied phenomenon.

Before interpreting the results, the assumptions of the analysis were verified. The distribution of residuals showed conformity with normal distribution, and analysis of the scatterplot of residuals versus predicted values confirmed homoscedasticity (constant error variance; MSresidual = 0.026). The absence of outliers and the analysis of predictor multicollinearity confirmed model stability. VIF values ranged from 1.12 to 2.38, indicating the absence of multicollinearity issues and enabling a reliable assessment of the unique contribution of each variable. VIF values above 2 for two predictors (anxiety and stress) were considered acceptable, given the strong theoretical justification for including both variables in the model and the absence of signs of estimator instability.

The constructed model showed a high statistical significance (F (10,96) = 10.58; *p* < 0.001), demonstrating a strong relationship between the set of predictors and the dependent variable (R = 0.72). The coefficient of determination (R^2^) was 0.52, indicating that the model explains 52% of the total variance in patients’ quality of life. After adjusting for the number of variables and sample size, the adjusted coefficient of determination was Radj^2^ = 0.4748, indicating a very high predictive ability of the model in the context of medical research. Table 21 shows the results of the model.

**Figure 1:**
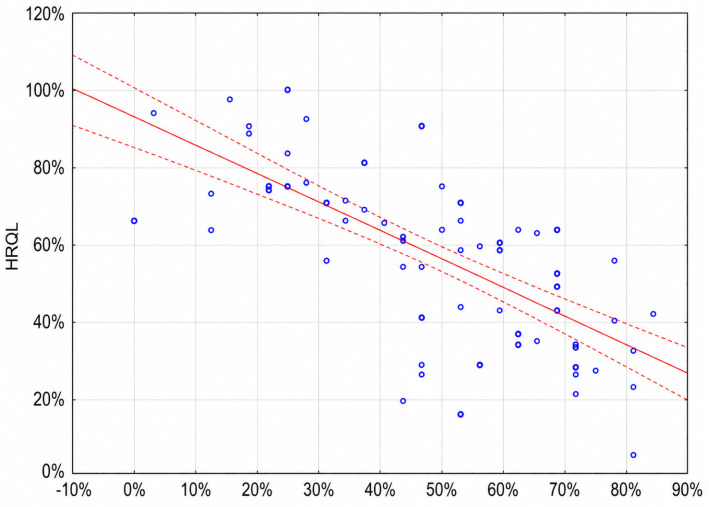
Scatterplot of HRQL versus symptom severity (%). The solid line represents the fitted regression line, and the dashed lines indicate the 95% confidence interval. The results clearly indicate that clinical factors are the primary determinants of quality of life in the studied group, with sociodemographic variables playing a secondary role.

The analysis of model parameters revealed a significant predictor of HRQL. Symptom severity was identified as a significant determinant of quality of life (β = −0.667; *p* < 0.001), which showed the strongest effect size (η^2^ = 0.45) and maximal statistical power (1.00). The value of the unstandardized coefficient (B = −0.71; 95% CI: −0.82 to −0.52) indicated that each one-unit increase in symptom severity was associated with an average decrease in HRQL of 0.71 points, assuming that all other variables remained constant. This implies that this variable alone accounts for approximately 45% of the unique variance in quality of life (Figure 1).

The remaining variables (age, education, place of residence, marital status, body weight, history of childbirth, anxiety, stress, and BMI) did not reach statistical significance, and their effects were small (η^2^ < 0.03), suggesting their limited impact on HRQL in the presence of clinical factors.

## 4. Discussion

The present study demonstrated that symptom severity is the primary determinant of health-related quality of life (HRQL) in women with uterine fibroids. In the multivariate analysis, symptom severity was the only independent predictor of HRQL, whereas anxiety, stress, and most sociodemographic variables were not independently associated with overall quality of life. These findings are consistent with previous reports indicating that the physical burden of uterine fibroids has the greatest impact on daily functioning and well-being [1,2,3,5]. Participants reported moderate impairment in HRQL, with the greatest burden observed in the domains of concerns and sexual functioning. Symptoms such as heavy menstrual bleeding, pelvic pain, and pressure-related complaints may adversely affect not only physical functioning but also emotional and social well-being. Similar relationships between symptom burden and reduced HRQL have been reported in studies using the UFS-QoL questionnaire and other patient-reported outcome measures [7,8,9,10]. Collectively, these findings reinforce symptom control as the primary therapeutic goal in the management of uterine fibroids.

These results highlight the importance of assessing sexual functioning as a distinct component of patient well-being, particularly among women awaiting surgical treatment.

Interestingly, symptom severity was not significantly associated with anxiety levels. This observation suggests that preoperative anxiety may be driven less by the severity of fibroid-related symptoms and more by concerns related to surgery, hospitalization, anesthesia, fertility, body image, and future health. Previous studies have similarly demonstrated that anxiety associated with planned surgical treatment may occur independently of disease severity [10,11,12,13]. An important finding of the present study was the high prevalence of preoperative anxiety, with more than 40% of participants meeting the threshold for clinically significant anxiety. Despite this, anxiety and stress were not independently associated with overall HRQL. However, both factors were significantly related to poorer sexual functioning, suggesting that the intimate dimension of quality of life may be particularly sensitive to emotional distress. Comparable findings have been reported in women with other gynecological conditions, including endometriosis and chronic pelvic pain disorders [14,15,16]. The substantial need for medical information observed in our study further supports the notion that uncertainty and perceived lack of control may contribute to psychological distress. Therefore, structured preoperative counseling and effective physician–patient communication may represent important components of comprehensive care.

Most sociodemographic factors, including age, marital status, education, and parity, were not significantly associated with HRQL. Nevertheless, women living in rural areas reported greater symptom severity and poorer quality of life than women residing in urban settings. These findings may reflect disparities in healthcare accessibility, delayed diagnosis, or reduced availability of specialist gynecological services in non-urban regions [17,18]. In addition, overweight participants reported poorer outcomes in selected domains, particularly concerns and sexual functioning, suggesting that metabolic and lifestyle-related factors may further influence subjective well-being [19].

Beyond their impact on physical and emotional well-being, uterine fibroids may substantially affect everyday functioning, work productivity, and social participation [20,21,22]. Previous studies have shown that women with symptomatic fibroids frequently report limitations in occupational performance, absenteeism, reduced work efficiency, and restrictions in daily activities, particularly when symptoms such as heavy menstrual bleeding and chronic pelvic pain are present [23,24]. Although work productivity was not directly assessed in the present study, the observed association between symptom burden and reduced HRQL supports the notion that the consequences of uterine fibroids extend beyond clinical symptoms alone. Future studies should investigate the impact of symptom severity on occupational functioning and socioeconomic outcomes to better characterize the overall burden of the disease. The increasing emphasis on patient-centered care has led to greater recognition of HRQL as a key outcome measure in uterine fibroid management. Recent guidelines and expert opinions suggest that treatment decisions should not be based solely on objective clinical parameters, such as fibroid size or number, but should also consider the patient’s symptoms, functional limitations, reproductive goals, and perceived quality of life [25]. The present findings support this approach by demonstrating that symptom burden, rather than demographic characteristics or psychological factors alone, is the principal determinant of HRQL.

From a clinical perspective, the present findings emphasize the need for a comprehensive and patient-centered approach to uterine fibroid management. Although effective reduction in symptom burden should remain the primary treatment objective, psychological well-being should also be routinely considered. While anxiety and stress did not independently predict overall HRQL, their association with impaired sexual functioning and their high prevalence among women awaiting surgery suggest that psychological support may improve important aspects of patient functioning and treatment experience. Integrating patient-reported outcomes with systematic assessment of emotional well-being may therefore contribute to more holistic and effective gynecological care.

### 4.1. Strengths and Limitations

Several limitations should be acknowledged. The relatively small sample size may have limited the power to detect weaker associations. In addition, the cross-sectional design precludes causal inference and does not allow changes in anxiety, stress, or quality of life to be assessed over time. The use of self-reported instruments may also have introduced response bias, particularly in sensitive areas such as sexual functioning. Finally, objective clinical variables such as fibroid size, location, and laboratory findings were not included in the analysis. Despite these limitations, the study was based on validated instruments and offers a comprehensive assessment of both somatic and psychological determinants of HRQL in women with uterine fibroids.

Another limitation is the lack of assessment of depressive symptoms. Since depression frequently coexists with anxiety and may influence health-related quality of life, future studies should include validated measures of depression to provide a more comprehensive evaluation of psychological well-being in women with uterine fibroids.

### 4.2. Practical Conclusions

The findings of this study underscore the central role of symptom burden in shaping quality of life among women with uterine fibroids and support symptom reduction as the primary aim of treatment. At the same time, the results draw attention to the importance of addressing psychological well-being, particularly in relation to sexual functioning. Integrating psychological support, clear medical communication, and patient education into routine care may contribute to better therapeutic outcomes and improved patient satisfaction.

## 5. Conclusions

Symptom severity was the main determinant of HRQL in women with uterine fibroids. Anxiety and stress were associated with poorer sexual functioning but not with symptom severity, suggesting that psychological burden may be related more to the diagnosis and planned surgery than to disease intensity. High informational needs across educational levels emphasize the importance of structured preoperative counseling and effective physician–patient communication. Rural residence and higher body weight were associated with poorer quality-of-life outcomes. Optimal care should integrate symptom-oriented treatment with psychological and educational support.

## Figures and Tables

**Table 1:** Anthropometric measurements of the respondents.

	M ± SD	Min–Max	Me [Q1–Q3]	95% CI
Height	169.06 ± 7.18	153–190	168 [165–173]	167.68–170.43
Body weight	68.59 ± 10.94	46–100	68 [60–74]	66.49–70.69
BMI	24 ± 3.55	17.75–31.64	23.74 [21.22–26.78]	23.32–24.68

**Table 2:** Characteristics of the study group.

		*n*	%
Age	<45 years	50	46.73%
	>45 years	57	53.27%
Education	Vocational	8	7.48%
	Secondary	18	16.82%
	Bachelor’s degree	16	14.95%
	Master’s degree	65	60.75%
Place of residence	Rural area	35	32.71%
	Town (<50,000 inhabitants)	37	34.58%
	City (50,000–200,000 inhabitants)	10	9.35%
	City (200,000–500,000 inhabitants)	7	6.54%
	City > (500,000 inhabitants)	18	16.82%
Marital status	Single	29	27.10%
	Married	71	66.36%
	Divorced	4	3.74%
	Widowed	3	2.80%
Type of residence	Living independently	7	6.54%
	Living with others	100	93.46%
Occupational status	Manual work	15	14.02%
	Non-manual work	79	73.83%
	Sick leave	1	0.93%
	Disability pension	10	9.35%
	Retirement	2	1.87%
Body weight status	Underweight	1	0.93%
	Normal	71	66.36%
	Overweight	29	27.10%
	Obese	6	5.61%

**Table 3:** Obstetric history of the study participants.

		*n*	%
Number of deliveries	0	51	47.66%
	1	24	22.43%
	2	26	24.30%
	3	5	4.67%
	4	1	0.93%
Number of miscarriages	0	93	86.92%
	1	5	4.67%
	2	5	4.67%
	3	4	3.74%
Vaginal delivery	0	71	66.36%
	1	13	12.15%
	2	20	18.69%
	3	3	2.80%
Cesarean section	0	85	79.44%
	1	17	15.89%
	2	5	4.67%
Gynecological surgeries	Yes	24	23.30%
	No	79	76.70%
Non-gynecological surgeries	Yes	24	23.30%
	No	79	76.70%

**Table 4:** Additional clinical symptoms of the study participants.

		*n*	%
Dyspareunia	Yes	19	17.76%
	No	88	82.24%
Pain during defecation	Yes	20	18.69%
	No	87	81.31%
Pain during urination	Yes	1	0.93%
	No	106	99.07%
Daily pain occurrence	Yes	15	14.02%
	No	92	85.98%
Dysmenorrhea	Yes	33	30.84%
	No	74	69.16%

**Table 5:** Assessment of anxiety and information needs using the APAIS questionnaire.

	*n*	M ± SD	Min–Max	Me [Q1–Q3]	95% CI
Anxiety related to anesthesia	107	4.57 ± 1.64	2–10	4 [3–6]	4.26–4.89
Anxiety related to the procedure	107	5.1 ± 1.62	3–10	5 [4–6]	4.79–5.41

**Table 6:** Distribution of anxiety levels and information needs according to the APAIS questionnaire.

Variable	Category	*n*	%
APAIS Anxiety	Normal	63	58.88
APAIS Anxiety	Clinically significant	44	41.12
Information Need	Low	31	28.97
Information Need	Moderate	38	35.51
Information Need	High	38	35.51

**Table 7:** Descriptive statistics for VAS anxiety and stress.

Variable	*n*	Mean ± SD	Min–Max	95% CI
VAS Anxiety	107	4.66 ± 2.22	0–10	4.24–5.09
VAS Stress	107	4.82 ± 2.19	0–10	4.40–5.24

**Table 8:** Place of residence in relation to anxiety, stress, and need for information.

	Place of Residence						U	*p*	Rs
	Rural Area			City/Town					
	M ± SD	Min–Max	Me [Q1–Q3]	M ± SD	Min–Max	Me [Q1–Q3]			
I	4.14 ± 1.38	2–8	4 [3–5]	4.78 ± 1.73	2–10	5 [3–6]	987	0.06	−0.22
II	4.94 ± 1.83	3–9	4 [4–6]	5.18 ± 1.52	3–10	5 [4–6]	1069	0.19	−0.15
III	9.09 ± 2.96	5–16	8 [7–10]	9.96 ± 2.97	6–20	9 [8–11.5]	976	0.06	−0.23
IV	6.4 ± 1.56	4–9	6 [5–8]	5.82 ± 2.12	2–10	6 [4–7]	1019	0.10	0.19
V	5.14 ± 2.16	2–8	5 [3–7]	4.43 ± 2.23	0–10	5 [3–6]	1027	0.12	0.19
VI	5.34 ± 1.89	2–9	5 [4–7]	4.57 ± 2.29	0–10	4.5 [3–6]	997	0.08	0.21

I. Anxiety related to anesthesia; II. Anxiety related to the procedure; III. Anxiety; IV. Need for information; V. VAS Anxiety; VI. VAS Stress.

**Table 9:** Anxiety assessment.

		Anxiety Assessment		Fisher’s Exact Test (*p*)	ϕ
		Normal Range	Clinically Significant		
Age	<45 years	33 (66%)	17 (34%)	0.17	0.14
	>45 years	30 (52.63%)	27 (47.37%)		
Education	Higher	46 (56.79%)	35 (43.21%)	0.5	−0.07
	Other	17 (65.38%)	9 (34.62%)		
Place of residence	Rural area	25 (71.43%)	10 (28.57%)	0.09	0.18
	City/town	38 (52.78%)	34 (47.22%)		
Marital status	Married	45 (63.38%)	26 (36.62%)	0.22	0.13
	Unmarried	18 (50%)	18 (50%)		
Body weight status	Normal	44 (61.97%)	27 (38.03%)	0.40	0.10
	Overweight	18 (51.43%)	17 (48.57%)		
Childbirth	Yes	32 (57.14%)	24 (42.86%)	0.84	−0.04
	No	31 (60.78%)	20 (39.22%)		

**Table 10:** Assessment of information need.

		Assessment of Information Need			χ^2^	*p*	V
		Low	Moderate	High			
Age	<45 years	13 (26%)	19 (38%)	18 (36%)	0.46	0.80	0.07
	>45 years	18 (31.58%)	19 (33.33%)	20 (35.09%)			
Education	Higher	25 (30.86%)	26 (32.1%)	30 (37.04%)	1.72	0.42	0.13
	Other	6 (23.08%)	12 (46.15%)	8 (30.77%)			
Place of residence	Rural area	6 (17.14%)	14 (40%)	15 (42.86%)	3.60	0.17	0.18
	City/town	25 (34.72%)	24 (33.33%)	23 (31.94%)			
Marital status	Married	22 (30.99%)	27 (38.03%)	22 (30.99%)	1.89	0.39	0.13
	Unmarried	9 (25%)	11 (30.56%)	16 (44.44%)			
Body weight status	Normal	25 (35.21%)	24 (33.8%)	22 (30.99%)	3.81	0.15	0.19
	Overweight	6 (17.14%)	14 (40%)	15 (42.86%)			
Childbirth	Yes	20 (35.71%)	17 (30.36%)	19 (33.93%)	2.81	0.25	0.16
	No	11 (21.57%)	21 (41.18%)	19 (37.25%)			

**Table 11:** Descriptive statistics for symptom severity.

Variable	*n*	Mean ± SD	Min–Max	95% CI
Symptom severity (%)	107	48.60 ± 20.97	0–84.38	44.58–52.62

**Table 12:** Assessment of quality of life across different domains.

	M ± SD	Min–Max	Me [Q1–Q3]	95% CI
Concerns	51.92 ± 29.67	0–100	50 [25–80]	46.23–57.6
Activities	57.84 ± 24.78	3.57–100	64.29 [35.71–75]	53.1–62.59
Mood/Energy	60.35 ± 22.44	0–100	60.71 [46.43–78.57]	56.05–64.65
Sense of control	59.95 ± 23.25	5–100	65 [40–80]	55.5–64.41
Self-awareness	58.41 ± 28	8.33–100	58.33 [41.67–83.33]	53.04–63.78
Sexual functioning	51.52 ± 28.3	0–100	50 [25–75]	46.09–56.94

**Table 13:** Descriptive statistics for overall HRQL.

Variable	*n*	Mean ± SD	Min–Max	95% CI
HRQL (%)	107	57.41 ± 22.30	6.03–100	53.14–61.69

**Table 14:** Correlation coefficients between anxiety, stress, and need for information and quality of life and symptom severity.

	Anxiety Related to the Procedure	Anxiety Related to Anesthesia	Anxiety	Need for Information	VAS Anxiety	VAS Stress
Symptom severity	0.12	0.02	0.10	0.13	0.05	0.01
Concerns	0.03	−0.04	−0.02	−0.09	−0.03	−0.01
Activities	0.04	0.01	0.02	−0.12	0.07	0.03
Mood/Energy	0.06	0.07	0.06	−0.04	0.13	0.14
Sense of control	0.06	0.02	0.04	−0.08	0.01	0.02
Self-awareness	−0.09	−0.02	−0.07	−0.16	−0.04	−0.05
Sexual functioning	−0.14	0.07	−0.04	−0.08	−0.28 **	−0.28 **
HRQL	0.01	0.02	0.01	−0.12	0.01	0.01

** *p* < 0.01.

**Table 15:** Association between age and symptom severity and quality of life.

	Age						U	*p*	Rs
	<45 Years			>45 Years					
	M ± SD	Min–Max	Me [Q1–Q3]	M ± SD	Min–Max	Me [Q1–Q3]			
I	50.75 ± 19.56	18.75–81.25	57.81 [31.25–68.75]	46.71 ± 22.14	0–84.38	50 [37.5–68.75]	1271	0.34	−0.11
II	51.8 ± 30.98	0–100	47.5 [25–80]	52.02 ± 28.74	0–100	50 [30–80]	1401	0.88	0.02
III	56.57 ± 26.45	3.57–100	60.71 [35.71–75]	58.96 ± 23.39	21.43–100	64.29 [35.71–75]	1352	0.65	0.05
IV	59.71 ± 21.3	0–100	60.71 [46.43–75]	60.9 ± 23.57	17.86–96.43	57.14 [42.86–85.71]	1404	0.90	0.02
V	59.9 ± 24.65	5–100	65 [40–80]	60 ± 22.16	15–100	65 [45–75]	1416	0.96	−0.01
VI	55.83 ± 29.08	8.33–100	58.33 [33.33–75]	60.67 ± 27.08	8.33–100	58.33 [50–83.33]	1305	0.45	0.08
VII	53.75 ± 32.46	0–100	50 [25–87.5]	49.56 ± 24.2	0–100	50 [25–62.5]	1338	0.58	−0.06
VIII	56.81 ± 23.22	6.03–100	59.91 [35.34–71.55]	57.94 ± 21.66	16.38–97.41	62.93 [41.38–74.14]	1363	0.70	0.04

I. Symptom severity; II. Concerns; III. Activities; IV. Mood/Energy; V. Sense of control; VI. Self-awareness; VII. Sexual functioning; VIII. HRQL.

**Table 16:** Association between education and symptom severity and quality of life.

	Education						U	*p*	Rs
	Higher			Other					
	M ± SD	Min–Max	Me [Q1–Q3]	M ± SD	Min–Max	Me [Q1–Q3]			
I	47.3 ± 21.92	0–84.38	53.13 [28.13–68.75]	52.64 ± 17.45	21.88–81.25	54.69 [43.75–62.5]	921	0.34	−0.13
II	52.96 ± 30.25	0–100	55 [30–80]	48.65 ± 28.09	15–95	45 [20–80]	936	0.40	0.11
III	59.7 ± 24.79	3.57–100	64.29 [35.71–78.57]	52.06 ± 24.29	14.29–96.43	53.57 [25–64.29]	824	0.10	0.22
IV	59.52 ± 22.82	0–100	60.71 [42.86–75]	62.91 ± 21.43	14.29–92.86	58.93 [46.43–78.57]	988	0.64	−0.06
V	61.36 ± 23.77	5–100	65 [45–80]	55.58 ± 21.37	20–85	55 [30–75]	865	0.17	0.18
VI	60.39 ± 26.92	8.33–100	58.33 [50–83.33]	52.24 ± 30.87	8.33–100	54.17 [16.67–75]	875	0.19	0.17
VII	54.94 ± 27	0–100	50 [37.5–75]	40.87 ± 30.12	0–100	37.5 [12.5–62.5]	774	0.04	0.27
VIII	58.52 ± 22.71	6.03–100	63.79 [42.24–75]	53.95 ± 21.03	23.28–90.52	57.76 [37.07–74.14]	905	0.28	0.14

I. Symptom severity; II. Concerns; III. Activities; IV. Mood/Energy; V. Sense of control; VI. Self-awareness; VII. Sexual functioning; VIII. HRQL.

**Table 17:** Association between place of residence and symptom severity and quality of life.

	Place of Residence						U	*p*	Rs
	Rural Area			City/Town					
	M ± SD	Min–Max	Me [Q1–Q3]	M ± SD	Min–Max	Me [Q1–Q3]			
I	53.84 ± 21.68	0–78.13	56.25 [50–68.75]	46.05 ± 20.29	3.13–84.38	46.88 [26.56–62.5]	923	0.03	0.27
II	41.29 ± 23.71	0–100	50 [20–55]	57.08 ± 31.01	0–100	55 [35–85]	914	0.02	−0.28
III	49.39 ± 23.86	3.57–100	53.57 [32.14–71.43]	61.95 ± 24.32	3.57–100	64.29 [46.43–78.57]	909	0.02	−0.28
IV	53.06 ± 21.45	17.86–96.43	53.57 [35.71–71.43]	63.89 ± 22.19	0–100	60.71 [50–80.36]	873	0.01	−0.31
V	52.43 ± 24.86	10–100	65 [30–70]	63.61 ± 21.66	5–100	65 [55–80]	959	0.045	−0.24
VI	50.95 ± 28.85	8.33–100	50 [16.67–75]	62.04 ± 27.04	8.33–100	66.67 [41.67–83.33]	996	0.08	−0.21
VII	43.57 ± 22.15	0–100	50 [25–50]	55.38 ± 30.24	0–100	50 [25–81.25]	948	0.04	−0.25
VIII	49.16 ± 20.68	16.38–97.41	52.59 [29.31–66.38]	61.42 ± 22.09	6.03–100	62.07 [42.67–75]	854	0.01	−0.32

I. Symptom severity; II. Concerns; III. Activities; IV. Mood/Energy; V. Sense of control; VI. Self-awareness; VII. Sexual functioning; VIII. HRQL.

**Table 18:** Association of marital status with symptom severity and quality of life.

	Marital Status						U	*p*	Rs
	Married			Unmarried					
	M ± SD	Min–Max	Me [Q1–Q3]	M ± SD	Min–Max	Me [Q1–Q3]			
I	47.89 ± 21.28	0–84.38	50 [31.25–68.75]	50 ± 20.58	3.13–81.25	59.38 [32.81–62.5]	1204	0.63	0.06
II	51.9 ± 30.06	0–100	55 [25–85]	51.94 ± 29.28	5–100	45 [25–80]	1233	0.77	−0.04
III	57.19 ± 25.69	3.57–100	64.29 [32.14–75]	59.13 ± 23.17	14.29–96.43	53.57 [42.86–80.36]	1248	0.85	0.02
IV	58.25 ± 23.03	0–100	57.14 [42.86–75]	64.48 ± 20.91	14.29–96.43	60.71 [46.43–78.57]	1074	0.18	0.16
V	57.82 ± 24.3	5–100	65 [40–75]	64.17 ± 20.68	20–90	75 [52.5–80]	1085	0.20	0.15
VI	57.04 ± 27.55	8.33–100	58.33 [41.67–75]	61.11 ± 29.07	8.33–100	66.67 [37.5–83.33]	1180	0.52	0.08
VII	52.29 ± 25.03	0–100	50 [37.5–75]	50 ± 34.2	0–100	50 [25–87.5]	1189	0.55	−0.07
VIII	56.29 ± 22.92	6.03–100	62.07 [34.48–71.55]	59.63 ± 21.17	23.28–93.97	59.05 [41.38–81.03]	1196	0.59	0.06

I. Symptom severity; II. Concerns; III. Activities; IV. Mood/Energy; V. Sense of control; VI. Self-awareness; VII. Sexual functioning; VIII. HRQL.

**Table 19:** Association between body weight status and symptom severity and quality of life.

	Body Weight Status						U	*p*	Rs
	Normal			Overweight					
	M ± SD	Min–Max	Me [Q1–Q3]	M ± SD	Min–Max	Me [Q1–Q3]			
I	49.82 ± 19.92	3.13–84.38	53.13 [34.38–68.75]	45.89 ± 23.28	0–81.25	46.88 [25–68.75]	1150	0.53	0.07
II	55.7 ± 28.83	0–100	55 [35–80]	43.43 ± 30.09	0–100	40 [20–60]	947	0.047	0.24
III	60.51 ± 24.54	3.57–100	64.29 [35.71–78.57]	52.14 ± 24.94	3.57–100	53.57 [28.57–67.86]	972	0.07	0.22
IV	60.06 ± 21.39	14.29–96.43	60.71 [42.86–75]	61.43 ± 24.85	0–100	57.14 [50–85.71]	1197	0.76	−0.04
V	60.42 ± 22.34	10–100	65 [40–80]	59.14 ± 25.6	5–100	60 [35–80]	1177	0.66	0.05
VI	61.5 ± 29.15	8.33–100	66.67 [50–83.33]	51.67 ± 24.9	16.67–100	50 [33.33–66.67]	965	0.06	0.22
VII	55.63 ± 25.16	0–100	50 [37.5–75]	43.93 ± 32.84	0–100	25 [25–75]	953	0.049	0.23
VIII	59.32 ± 22.29	16.38–97.41	63.79 [37.07–75]	53.47 ± 22.45	6.03–100	54.31 [29.31–66.38]	1007	0.11	0.19

I. Symptom severity; II. Concerns; III. Activities; IV. Mood/Energy; V. Sense of control; VI. Self-awareness; VII. Sexual functioning; VIII. HRQL.

**Table 20:** Association between patients’ history of childbirth and symptom severity and quality of life.

	Childbirth						U	*p*	Rs
	Yes			No					
	M ± SD	Min–Max	Me [Q1–Q3]	M ± SD	Min–Max	Me [Q1–Q3]			
I	48.1 ± 21.63	0–81.25	48.44 [35.94–68.75]	49.14 ± 20.43	0–84.38	53.13 [28.13–62.5]	1402	0.87	−0.02
II	53.3 ± 27.95	5–100	55 [30–80]	50.39 ± 31.65	0–100	45 [25–80]	1326	0.53	0.07
III	59.25 ± 25.32	7.14–100	67.86 [32.14–78.57]	56.3 ± 24.32	3.57–100	53.57 [42.86–67.86]	1275	0.34	0.11
IV	59.57 ± 21.01	14.29–96.43	57.14 [44.64–76.79]	61.2 ± 24.09	0–100	60.71 [46.43–78.57]	1332	0.55	−0.07
V	61.07 ± 21.61	15–100	65 [45–80]	58.73 ± 25.08	5–100	60 [35–80]	1350	0.63	0.05
VI	59.97 ± 27.5	16.67–100	66.67 [41.67–83.33]	56.7 ± 28.72	8.33–100	58.33 [33.33–75]	1304	0.44	0.09
VII	51.12 ± 24.34	0–100	50 [37.5–68.75]	51.96 ± 32.34	0–100	50 [25–87.5]	1412	0.92	0.01
VIII	58.13 ± 21.13	19.83–97.41	63.79 [40.95–75]	56.63 ± 23.71	6.03–100	58.62 [37.07–71.55]	1329	0.54	0.07

I. Symptom severity; II. Concerns; III. Activities; IV. Mood/Energy; V. Sense of control; VI. Self-awareness; VII. Sexual functioning; VIII. HRQL.

**Table 21:** Results of multiple regression analysis for the dependent variable HRQL.

Predictor	B	SE	t	*p*	β	η^2^	Moc
Intercept (constant term)	1.04	0.19	5.59	<0.001		0.25	1.00
Age	0.00	0.00	0.31	0.76	0.02	0.00	0.06
BMI	−0.01	0.00	−1.83	0.07	−0.14	0.03	0.44
Need for information	0.00	0.01	0.01	1.00	0.00	0.00	0.05
Anxiety	0.00	0.01	0.56	0.57	0.06	0.00	0.09
VAS Stress	0.00	0.01	−0.14	0.89	−0.02	0.00	0.05
Symptom severity	−0.71	0.08	−8.89	<0.001	−0.67	0.45	1.00
Education	0.01	0.02	0.28	0.78	0.02	0.00	0.06
Place of residence	−0.03	0.02	−1.38	0.17	−0.13	0.02	0.28
Marital status	−0.01	0.02	−0.60	0.55	−0.05	0.00	0.09
Childbirth	0.01	0.02	0.49	0.62	0.04	0.00	0.08

B: unstandardized coefficient; SE: standard error; β: standardized coefficient; η^2^: partial eta squared. Model statistics: *n* = 107; R^2^ = 0.524; adjusted R^2^ = 0.475; F (10,96) = 10.58; *p* < 0.001.

## Data Availability

The data presented in this study are available on request from the corresponding author.
